# Immediate dental implant placement in post-extraction-infected sites decontaminated with Er,Cr:YSGG laser: a retrospective cohort study

**DOI:** 10.1007/s10266-022-00734-4

**Published:** 2022-09-08

**Authors:** Rolando Crippa, Riccardo Aiuto, Mario Dioguardi, Michele Nieri, María Peñarrocha-Diago, Miguel Peñarrocha-Diago, Francesca Angiero

**Affiliations:** 1 Department of Medical Sciences and Diagnostic Integrated, S. Martino Hospital, University of Genoa, Genova, Italy; 2 Stomatology Department, Faculty of Medicine and Dentistry, University of Valencia, Valencia, Spain; 3 Department of Biomedical, Surgical and Dental Sciences, University of Milan, Milan, Italy; 4 Department of Clinical and Experimental Medicine, University of Foggia, Foggia, Italy; 5 Department of Experimental and Clinical Medicine, University of Florence, Florence, Italy

**Keywords:** Dental implant, Er,Cr:YSGG laser treatment, Oral surgery, Tooth extraction, Socket preservation

## Abstract

Dental implants placed in fresh extraction alveoli provide several advantages, including shorter treatment periods and improved patient comfort. After a compromised tooth extraction, the Er,Cr:YSGG laser can considerably reduce bacterial concentration. The objective of this controlled study conducted after at least 1 year of follow-up was to compare the use of immediate post-extraction implants in infected sites treated with laser (test group) versus conventional implants in edentulous sites (control group) through an analysis of pre- and post-operative radiographs. The study was based on a series of patients treated between 2014 and 2019, with a 1-year minimum follow-up, and up to over 4 years. An analysis of the clinical history of the treated patients and pre- and post-operative radiographs was performed to evaluate the implant success and to measure the marginal bone level (MBL). Overall, 149 implants were studied. There was only one failure in the test group (1%) and no failures in the control group. The test group gained 0.1 mm of the MBL compared to the baseline, while the control group lost 0.1 mm of the MBL. The difference between the two groups of only 0.2 mm was not statistically significant (*P* = 0.058). Immediate dental implants in infected sockets debrided and decontaminated using Er,Cr:YSGG laser do not appear to enhance the likelihood of failure; however, peri-implantitis and associated problems must be avoided by following a certain set of protocols and procedures.

## Introduction

Immediate placement of a dental implant (type 1 placement technique [1]) has become a popular therapeutic choice in recent years. Schulte and Heimke introduced the surgical approach for immediate insertion of a fixture in a fresh alveolar socket in 1976 [2]. This protocol's proponents argue that by limiting the surgical exposure of the patient, bone resorption following dental extraction is decreased [3]. This technique has been effectively used to several forms of implant-prosthetic rehabilitation, and many scientific studies demonstrate its validity [4, 5]. Dental implants inserted in post-extraction sites have several advantages, including decreased treatment time and improved patient comfort [6].

Tooth extraction is frequently associated with an apical infection: one of the main limits to early implant placement is represented by a bacterial contamination of the implant surface during the healing process [7]. Animal studies, on the other hand, have shown that a periapical lesion does not limit the osseointegration of post-extraction fixtures. Furthermore, BIC (bone-to-implant contact) is not affected [8–12]. An increasing number of publications have detailed the feasibility of this dental implant technique also in infected dental alveoli, although dependent on whether the correct indicators are present and if a rigorous decontamination protocol is adhered to [13].

Various methods for decontaminating the post-extraction site prior to implant placement have been described. Marconcini et al. proposed tooth extraction with utmost caution to maintain alveolar bone integrity, as well as delicate curettage of sockets [14]. Besides, antibiotics and chlorhexidine mouth rinses are two strategies for reducing the bacterial load of infected alveoli. Del Fabbro et al. published a similar approach in a cohort study, but with the inclusion of PRGF in infected alveoli [15]. Lasers have also been added to the clinical protocol to obtain thorough decontamination and to limit case failures. Kusek presented the first case series, which included 10 immediate implantation [16]. Later, other authors carried out independent research illustrating the use of Er,Cr:YSGG laser to treat dental alveoli [17–19].

The objective of this controlled study, conducted within at least 1 year of follow-up after treatment was to compare the use of immediate implants (type 1) in post-extraction-infected alveoli debrided and decontaminated with Er,Cr:YSGG laser (test) versus conventional implants in edentulous sites (control) in a sample of treated patients. The primary variable was the difference in MBL (marginal bone level) between the follow-up and baseline (implant placement). The outcome variables included implant failure and complications (such as mucositis or peri-implantitis).

## Materials and methods

### Study design

This research received the approval of the ethics committee of the University of Valencia (no. 1606937298573) and was performed in strict compliance with the STROBE statement (von Elm et al. 2008). The current study was based on a series of patients treated between 2014 and 2019, with a 1-year minimum follow-up, up to over 4 years (the calculation of the sample size was not necessary as all patients were included in the time period indicated). The current study was carried out in collaboration with the Istituto Stomatologico Italiano of Milan (Italy) at the Oral Surgery Department of the University of Valencia (Spain).

The first step of this study included the selection of the X-rays (intraoral periapical radiograph taken with the positioning ring and the parallel technique) and the medical records of the included patients. All participants either received immediate dental implant treatment placed in infected alveoli debrided and decontaminated with lasers or received implants using traditional techniques. Patients must have had a minimum of 1-year follow-up. Other exclusion criteria were as follows: participants with significant systemic disorders, history of radiation therapy, current steroid treatment, neurological or psychiatric problems, immunocompromised status, bruxism, a smoking habit (more than 15 cigarettes per day), alcohol or drug use, and poor compliance.

**Fig. 1:**
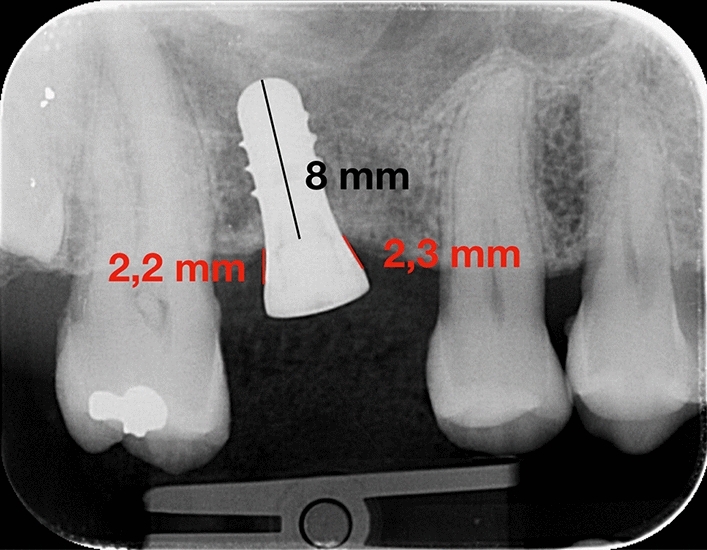
Example of X-ray measurement for MBL: 8 mm was the length of the implant used for calibration, while 2.3 and 2.2 mm show the mesial and distal MBL measurement, respectively

The second step of this study included the measurement of digital radiographs by a blinded operator (R.A.) with a specific software (Image J, National Institute of Health, Bethesda, Rockville, MA, USA). The following parameters were used for radiographs: 65–90 kV, 7.5–10 mA, and 0.22–0.25 s. Each periapical X-ray was calibrated prior to examination by considering the parameters of the fixture (diameter and length) as reference values to adjust for any distortion. The radiographs were measured on a medical screen with a resolution of 1920 × 1080 and with magnification of 7x. Marginal bone level was quantified at baseline and follow-up according to Linkevicius et al. The segment between the fixture neck and the first bone-to-implant contact was calculated and taking into consideration both the mesial and distal parts of each fixture (Fig. 1).

An intra-rater agreement was performed for the radiographic evaluation. An a-priori independent sample of 20 fixture surfaces was assessed twice, 2 weeks apart. For radiographic intra-examiner agreement test, the two-way intra-class correlation coefficient was 0.97 (95% CI).

### Statistical analysis

As descriptive statistics, the mean and standard deviation of the quantitative variables, as well as the frequency and percentages of the qualitative variables, were utilized. The implant was the subject of analysis; accounting for the fact that multiple implants were often used for each patient.

A mixed statistical model was used for the outcome variable difference in MBL using the patient as a random effect. The covariate was the MBL at baseline, and the group (test or control implant) was the explanatory variable (fixed effect).

To compare the contrast at baseline between the two groups (test implants versus control implants), mixed-effects models were used for the quantitative variables including age, implant length, implant diameter, and MBL at baseline. A mixed-effects model was also used to compare the duration before a follow-up between the two groups. The participant was the random effect (random effect), and the group (implant test or control) was the explanatory variable (fixed effect).

To compare the differences at baseline between the two groups, multilevel models were used for the qualitative variables: sex, smoking, arch (upper or lower), area (frontal—incisors or canines—or posterior—premolar or molar), extraction reasons (fracture vs. other), presence of abscess or fistula, presence of lesion, implants with narrow neck, immediate loading, use of membrane, use of collagen, and use of synthetic bone. The models were constructed at two levels (patient and implant), and the group (test implant or control) was the explanatory variable. The significance level was set at *P* < 0.05; statistical analysis was carried out using JMP v. 13.0, and MLwin v. 3.05.

### Surgical phase

All patients consented to a therapeutic plan including the dental extraction, debridement and decontamination of the alveoli using the Er,Cr:YSGG 2780 nm laser (for all surgical phases), and insertion of a fixture in the same appointment, to replace the extracted tooth (test group). The treatment plan was decided following a thorough examination that ruled out any contraindications. The patients provided informed consent for data processing.

**Fig. 2:**
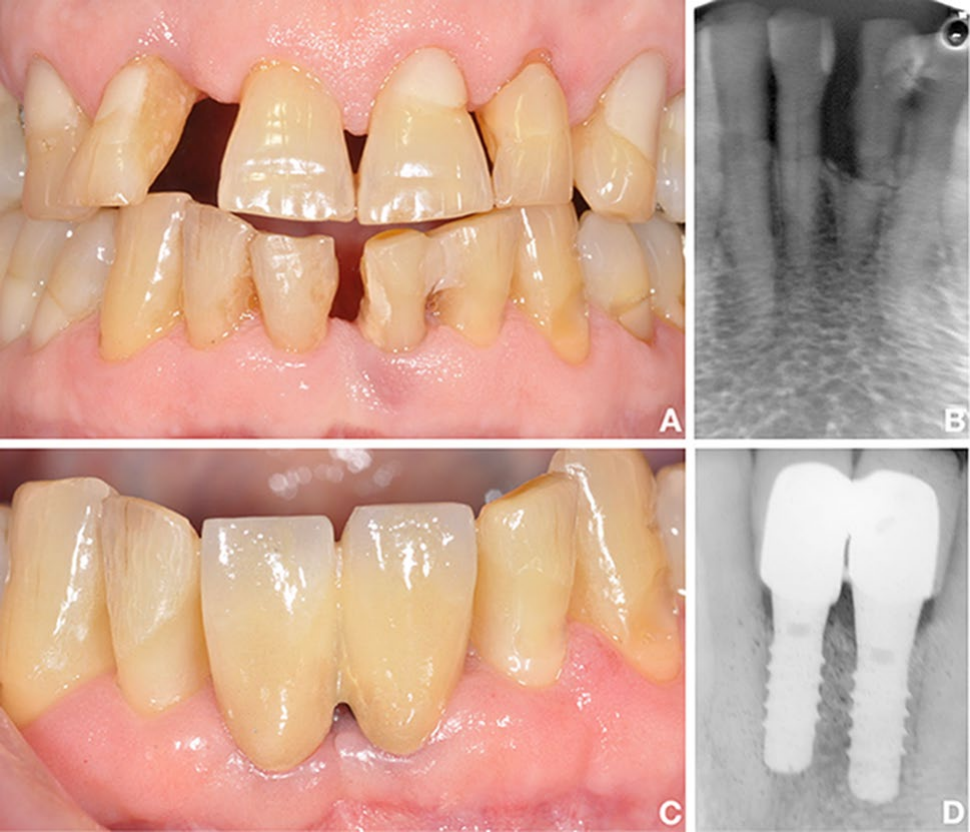
Pre-operative (**a**, **b**) and post-operative (**c**, **d**) clinical and radiological conditions of a case of the analyzed sample: the fractured teeth 3.1 and 4.1 were replaced with two post-extraction implants (test group)

**Fig. 3:**
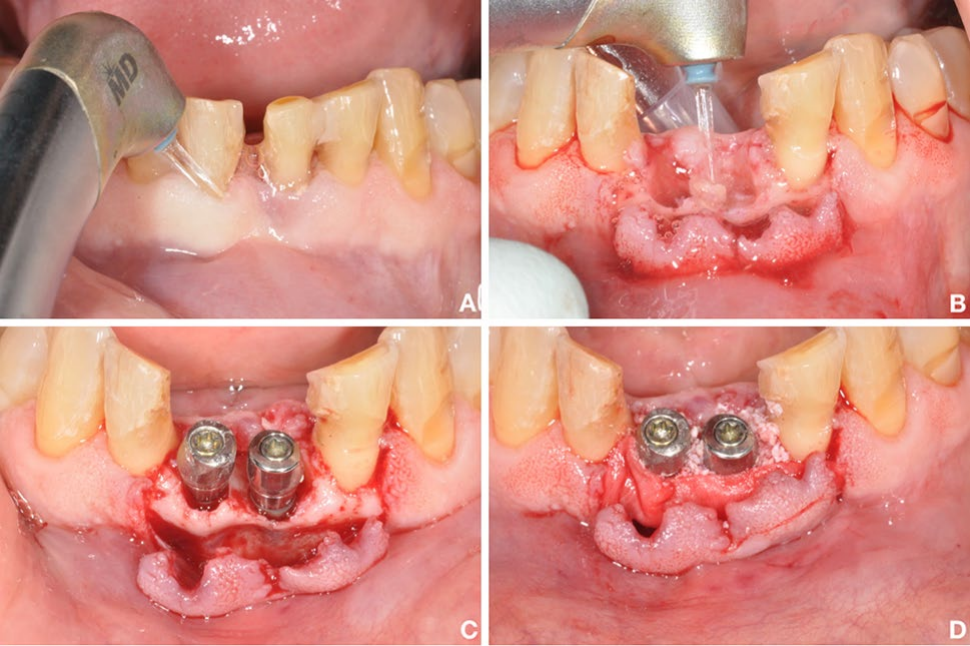
Some phases of surgery (test group) that include the application of the laser for atraumatic extraction (**a**) and site disinfection (**b**), the placement of the fixtures (**c**), and the use of biomaterials (**d**)

**Fig. 4:**
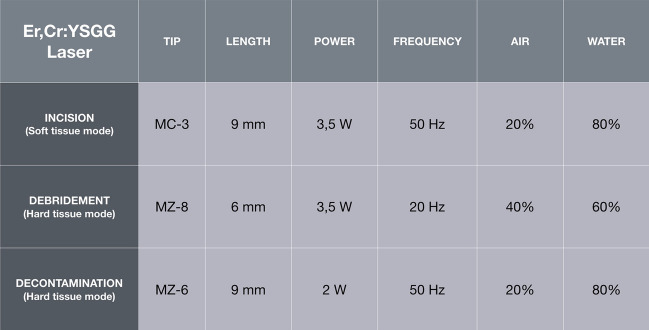
Laser parameters

The surgical phase (Figs. 2 and 3) included antibiotic prophylaxis that started the night before intervention. The local anesthetic administered in the interventions was Optocain^®^. The compromised teeth were extracted atraumatically to conserve the remaining tissues. The flap was performed by the laser with specific parameters: settings for the soft-tissue mode (s), which included an MC-3 tip at a length of 9 mm, including 20% air and 80% water. The tip was in contact with the tissue, simulating the action of the scalpel (chisel tip), and it was used with soft-tissue parameters. Once the extraction was carried out, the debridement phase of the infected alveolus has begun. For bone tissue, the parameters included an MZ-8 tip at a length of 6 mm, including 40% air and 60% water. The site was decontaminated with the hard tissue mode (H), 2.0 W, 20% air, and 80% water, while mounting a 9 mm MZ-6 tip. To reach the apex of the dental socket more easily, the tip was changed again; during the decontamination phase, the tip was not in contact, but approached the bone 1-2 mm approximately. The laser was the only tool used to remove infected tissue (Fig. 4). Debridement time was determined by the bone volume and amount of pathological tissue (it is a mechanical action performed exclusively with the laser), while decontamination lasted from 60 to 90 s per alveolus (wash out H2O 100 ml/min), without contact between the tip of the laser and the bone (it is a bactericidal action that exploits the photoacoustic effect of the laser). All laser treatments were performed with the Waterlase iPlus® (Biolase) equipment.

The following phases of the surgery included the placement of the implants (Straumann^®^). Often, it is also essential to put in place biomaterials for the infection-related residual defects. Collagen (Septodont^®^) and an absorbable membrane (Collprotect^®^) were applied to promote tissue repair. Sutures were carefully inserted to provide optimal flap repositioning. Subsequently, 0.2% chlorhexidine gluconate gel was prescribed for 2 weeks, and post-operative instructions were illustrated to the patient. The fixtures were either loaded immediately or after 3 months.

Patients in the control group followed a similar implant protocol, but tooth extraction had taken place at least 3 months earlier. There was no laser debridement/decontamination of the site.

## Results

Overall, 98 patients aged 58.0 ± 14.6 years (21–88 years), 52 females (53%) and 46 males (47%), 22 smokers (22%) were treated. Of which, 149 implants were placed for 90 (60%) test subjects and 59 (40%) control subjects.

Test implants were placed in 53 patients (one fixture was inserted in 35 patients, two simultaneous implants were inserted in 10 patients, three simultaneous implants in four patients, four simultaneous implants in three participants, and five simultaneous implants in one patient). Control implants were placed in 39 patients (one fixture was inserted in 29 patients, two simultaneous implants were inserted in seven patients, and three simultaneous implants in three patients). Both experimental and control implants were inserted in six patients (one test implant and one control implant in five patients, and one test implant and two control implants in one patient).

### Baseline

Patient-related variables at baseline are shown in Table 1. The table refers to patients who had at least one implant of either type. The variables related to the site are listed in Table 2.

**Table 1:** Patient-related baseline characteristics

Variable	Test group (*N* = 59)	Control group (*N* = 45)	*P* value
Sex (female) (%)	29 (49%)	25 (56%)	0.764*
Sex (male) (%)	30 (51%)	20 (44%)	0.764*
Age (years) (SD)	59.3 (14.5)	57.5 (14.5)	0.977**
Smoker (%)	13 (22%)	9 (20%)	0.913*

*SD* standard deviation

*Multilevel model

**Mixed model

**Table 2:** Baseline characteristics related to the implant

Variable	Test group (*N* = 90)	Control group (*N* = 59)	*P* value
Upper arch	47 (52%)	25 (42%)	0.279*
Lower arch	43 (48%)	34 (58%)	0.279*
Zone (anterior)	26 (29%)	9 (15%)	0.201*
Zone (posterior)	64 (71%)	50 (85%)	0.201*
Extraction (fracture)	43 (48%)	31 (52%)	0.987*
Extraction (no fracture)	47 (52%)	28 (48%)	0.987*
Abscess or fistula	61 (68%)	42 (72%)	0.866*
Lesion	20 (22%)	2 (3%)	0.007*
Narrow neck	12 (13%)	10 (17%)	0.563*
Implant length, mm (SD)	9.9 (1.7)	8.9 (1.7)	0.001**
Implant diameter, mm (SD)	3.9 (0.4)	4.0 (0.5)	0.232**
Immediate loading	21 (23%)	8 (14%)	0.534*
Membrane	69 (77%)	30 (51%)	0.047*
Collagen	21 (23%)	22 (37%)	0.324*
Synthetic bone	55 (61%)	18 (31%)	0.011*
MBL baseline mm (SD)	2.4 (1.3)	2.4 (0.8)	0.912**

*SD* standard deviation

*Multilevel model

**Mixed model

In the test group, lesions were more common. Additionally, if the implant length was greater than 1 mm, the membrane and synthetic bone were more frequently used. The reasons for extraction in the test group included: caries 32 (36%), endodontic 10 (11%), fracture 43 (48%), and periodontal 5 (6%); and in the control group: caries 20 (34%), endodontic 1 (2%), fracture 31 (52%), and periodontal 7 (12%).

The implants were all Straumann implants. In the test group, TE RN Loxim SLA Roxolid 33 (37%), S RN Loxim SLA Roxolid 37 (41%), SP RN Loxim SLA TiZr 4 (4%), SP NNC SLAactive TiZr 3 (3%), S RN SLAactive Roxolid 4 (4%), and SP NNC Loxim SLA Roxolid 9 (10%). In the control group, TE RN Loxim SLA Roxolid 6 (10%), S RN Loxim SLA Roxolid 39 (66%), SP RN Loxim SLA TiZr 4 (7%), SP NNC SLAactive TiZr 0 (0%), S RN SLAactive Roxolid 0 (0%), and SP NNC Loxim SLA Roxolid 10 (17%).

### Follow-up

The follow-up was carried out after 1.7 ± 0.6 years in the experimental group and 1.5 ± 0.5 years in the control group, with a non-statistically significant difference (*P* = 0.082; Mixed model). There was only one failure in the test group (1%) and no failure in the control group. There was only one complication (mucositis) in the control group (2%) and no complications other than failure in the test group. MBL results at follow-up are shown in Table 3.

**Table 3:** Marginal bone level (MBL) at follow-up

Variable	Group test (*N* = 89)	Group control (*N* = 59)	Diff	95% CI	*P* value
MBL at follow-up, mm (SD)	2.3 (0.9)	2.5 (0.7)	0.2	0.0; 0.4	0.058*
MBL difference between baseline and follow-up, mm (SD)	0.1 (1.0)	− 0.1 (0.6)	0.2	0.0; 0.4	0.058*

*SD* standard deviation

*Mixed model

The difference in MBL between the two groups was in favor of the experimental group which gained 0.1 mm relevant to the baseline while the control group lost 0.1 mm of MBL. However, the difference between the two groups was only 0.2 mm, which was not statistically significant (*P* = 0.058).

## Discussion

From the results, the two groups appeared sufficiently homogeneous in terms of patient's age and gender and the areas treated, making the comparison of this retrospective study more reliable. Surgical options, such as implant length or the use of biomaterials, often vary according to the clinical situation. For example, in the test group, the implants were longer. In addition, membranes and autologous bones were used more often in the test group, since lesions were detected more often, and thus, bone defects were treated more frequently.

The main objective of this research was to compare post-extraction implants in infected sites to the traditional technique, where fixtures were placed at least 3 months after extraction and without signs of residual infections in the alveoli. The results indicated that there was no difference in MBL between the two groups. Since it is not always easy to identify the presence of an active infection when it is necessary to remove a compromised tooth, type 4 implants were chosen for the control group. They were positioned in edentulous areas with good healing of the post-extraction socket. Therefore, in this situation, we can be sure that surgery was performed in an edentulous area free of bacteria.

This study analyzed 149 implants in total, with mesial and distal MBL measurements at baseline and follow-up. To the best of our knowledge, this is the only controlled study in the literature on implant insertion in infected alveoli debrided and decontaminated with the Er,Cr:YSGG 2780 nm laser. In a recent meta-analysis, Lee et al. showed the same encouraging conclusion by analyzing five prospective studies that did not involve the use of laser but more conventional debridement techniques; the same authors reported the absence of RCTs on the topic in the literature [20].

In a recent publication by Kakar et al., the authors followed a clinical protocol similar to the present study, including debridement with Er,Cr:YSGG 2780 nm laser, to treat a case series without a control group [19]. However, despite the lack of measurements of the MBL, the highlight of the study was that the survival of the implants exceeded 95%, which is in line with the survival rate expected from conventional implantology methods.

Evaluating the success of implant therapy, it is important to calculate the MBL, of which up to 2 mm can be considered as physiologic bone remodeling [21]. The data obtained on the MBL in this research are not only in line with or lower than our control group, but it is also comparable to that of other studies. Among these, Berberi et al. described the MBL in immediate and delayed loading techniques of post-extraction implants [22]; immediate loading seems to guarantee promising clinical results, as shown by several cases in this study.

Previous research comparing panoramic and periapical radiographs found that the latter is deemed the “gold standard” for detecting implants’ MBL [23, 24]. CBCT would also be useful, but due to the dose of rays and lack of justification, it would not be possible to find a sufficient number of patients for the study. The need to have comparable radiographs has led to a scrupulous selection of patients to increase the reliability of the data. This could be a limitation of the present study. Another limitation of this work was the modest number of implants losses, making the random-effects logistic regression analysis unmeaningful, and hence, the potential predictors recorded herein could not be related to early or late fixture loss. Furthermore, it is a retrospective study, which implies the presence of some bias, albeit with a protocol already published in the previous studies by the same authors [18].

Candidate selection is crucial to the protocol's effectiveness. Some rules, in the authors' opinion, must be followed. First, the patient must be in good health, possibly a nonsmoker, and not have untreated periodontal disease. The candidate must be cooperative and follow the dentist's indications. Second, the clinical situation should be meticulously evaluated in advance, including the reason for tooth extraction, the occurrence of recurrent infections, and the kind of bone. As a result, radiographs and, if applicable, CBCT must be evaluated. Third, surgical prophylaxis, which includes antibiotic medication and 0.2% chlorhexidine gel, must be provided. Fourth, to preserve the leftover bone, the extraction must be conducted atraumatically. Fifth, among the different types of lasers, Er,Cr:YSGG is indicated for the good decontamination capacity without overheating the surrounding bone [25]. Finally, the application of biomaterials is frequently required to deal with bone defects and must be included.

The laser was introduced into dental practice by Leon Goldman in 1964. The erbium wavelengths in mid-IR spectra have high affinity for HA (hydroxyapatite) and water. Because of the high affinity for water, the penetration depth is minimal, allowing for good surface ablation without harming the deep tissues. Erbium lasers may cut soft tissues and bone with minimum heat damage, in favor of less inflammatory reactions and faster recovery [26]. The use of Er,Cr:YSGG lasers in dental practice has been widely researched and applied in a variety of applications. Their application as an adjuvant to standard periodontal therapy, for example, has been shown to be successful in bacterial reduction. Furthermore, as demonstrated by Dereci et al., Er,Cr:YSGG lasers are effective in the coagulation of opening blood vessels and the de-epithelization of the gingival pocket [27]; however, in these cases, the hemostatic action is mainly due to the surgical toilet and the removal of the granulation tissue with the laser. ER,Cr:YSGG lasers have also been shown to improve cell adhesion and migration on root surfaces [28]. The Er,Cr:YSGG laser has been shown to be a useful tool in endodontic therapy: Martins et al. proved that a laser-assisted approach, thanks to the photoacoustic effect, is efficient against a wide range of pathogens [29].

Regardless of the demonstrated laser decontamination action, multiple investigations have shown that if specific safeguards are performed, immediate implants can also be inserted in contaminated sites. Waasdorp et al. confirmed in a comprehensive study that sites must be extensively debrided before to placement, and GBR is typically conducted to cover the gaps between the implant and socket [30]. This dental implant procedure, definitely, has a learning curve and necessitates prior implantology experience. There are some drawbacks, such as the device's price. A review of the trials on the topic shows that immediate dental implants into contaminated sites do not raise the rate of problems or impede tissue integration, as long as correct clinical protocols are followed to obtain a good alveolus cleaning [31].

Plaque accumulation is the primary cause of periodontitis, and the progression from periodontitis to peri-implantitis happens in the absence of supporting maintenance therapy [32]. Pre-operative antibiotic usage reduces implant failures, according to Dent et al. [33]. Nonetheless, a systematic review concludes that the advantages of antibiotic administration for non-infected alveoli are uncertain and may be unnecessary [34, 35]. It is also crucial to note that the existence of some systemic conditions or dangerous habits (i.e., smoking) and local risk factors (i.e., presence of keratinized tissue or type of implant surface) may enhance the risk of peri-implantitis [36].

In this implant placement protocol, the authors followed current surgical protocols that include antibiotic prophylaxis, also to prevent systemic superinfections such as bacterial endocarditis, and chlorhexidine in the post-operative period. Consequently, it is not possible to establish a clear causal effect of the laser alone on decontamination and implant success. In any case, the aim of the work is to show the clinical and radiographic success of fixtures placed in infected sites, highlighting a percentage of failure comparable to that of the traditional method and a total healing of osteolytic lesions where present. Therefore, further prospective clinical trails, preferably randomized, are needed to enlighten these aspects. For example, a randomized study (RCT), possibly with a split-mouth design, comparing immediate and non-immediate implants placement in infected sites would be helpful to understand the percentage of success of the first technique versus the second one. Regardless, the described technique is based on recent scientific knowledge and clinical practice that encourages dental implant type 1, even in post-extraction-infected alveoli.

It is quite complex to draw conclusions from this study. In fact, as a non-randomized study, it is difficult to establish to what extent the differences obtained in the two groups were due to the therapy or to the presence of patients, sites, and implants with different characteristics in the two groups. Implants were longer in the test group. In addition, membranes and autologous bones were used more often in the test group. However, the result that there is no difference in MBL, which was improved in the test group, seems promising for the clinical application of the described technique for immediate dental implants’ insertion in infected alveoli. This technique has various advantages, such as a decreased time of the clinical session and a higher patient comfort, and it does not seem to raise the risk of failure, but it is crucial to follow several precautions and certain procedures to prevent complications like peri-implantitis. This is precisely the most significant conclusion, namely the fact of being able to have a less-invasive surgery, with shorter clinical and biological times and without an increased risk of losing the implant.

## References

[1] Hammerle CH, Chen ST, Wilson TG (2004). Consensus statements and recommended clinical procedures regarding the placement of implants in extraction sockets. Int J Oral Maxillofac Implants.

[2] Schulte W, Heimke G (1976). The Tubinger immediate implant. Quintessenz.

[3] Paolantonio M, Dolci M, Scarano A, d’Archivio D, di Placido G, Tumini V (2001). Immediate implantation in fresh extraction sockets. A controlled clinical and histological study in man. J Periodontol.

[4] Rodrigo D, Martin C, Sanz M (2012). Biological complications and peri-implant clinical and radiographic changes at immediately placed dental implants. A prospective 5-year cohort study. Clin Oral Implants Res.

[5] Peñarrocha-Diago M, Maestre-Ferrín L, Demarchi CL, Peñarrocha-Oltra D, Peñarrocha-Diago M (2011). Immediate versus nonimmediate placement of implants for full-arch fixed restorations: a preliminary study. Oral Maxillofac Surg.

[6] Koh RU, Rudek I, Wang HL (2010). Immediate implant placement: positives and negatives. Implant Dent.

[7] Schwartz-Arad D, Chaushu G (1997). The ways and wherefores of immediate placement of implants into fresh extraction sites: a literature review. J Periodontol.

[8] Chang S-W, Shin S-Y, Hong J-R (2009). Immediate implant placement into infected and noninfected extraction sockets: a pilot study. Oral Surg Oral Med Oral Pathol Oral Radiol Endod.

[9] Novaes A, Marcaccini A, Souza S, Taba MJ, Grisi MF (2003). Immediate placement of implants into periodontally infected sites in dogs: a histomorphometric study of bone-implant contact. Int J Oral Maxillofac Implants.

[10] Marcaccini M, Novaes AJ, Souza S, Taba MJ, Grisi MF (2003). Immediate placement of implants into periodontally infected sites in dogs. Part 2: a fluorescence microscopy study. Int J Oral Maxillofac Implants.

[11] Novaes AJ, Papalexiou V, Grisi M, Souza SS, Taba M, Kajiwara JK (2004). Influence of implant microstructure on the osseointe- gration of immediate implants placed in periodontally infected sites. A histomorphometric study in dogs. Clin Oral Implants Res.

[12] Papalexiou V, Novaes A, Grisi M, Souza SS, Taba M, Kajiwara JK (2004). Influence of implant microstructure on the dynamics of bone healing around immediate implants placed into periodontally infected sites. A confocal laser scanning microscopic study. Clin Oral Implants Res.

[13] Corbella S, Taschieri S, Tsesis I, Del Fabbro M (2013). Postextraction implant in sites with endodontic infection as an alternative to endodontic retreatment: a review of literature. J Oral Implantol.

[14] Marconcini S, Barone A, Gelpi F, Briguglio F, Covani U (2013). Immediate implant placement in infected sites: a case series. J Periodontol.

[15] Del Fabbro M, Boggian C, Taschieri S (2009). Immediate implant placement into fresh extraction sites with chronic periapical pathologic features combined with plasma rich in growth factors: preliminary results of a single-cohort study. J Oral Maxillofac Surg.

[16] Kusek ER (2011). Immediate implant placement into infected sites: bacterial studies of the Hydroacoustic effects of the YSGG laser. J Oral Implantol.

[17] Montoya-Salazar V, Castillo-Oyagüe R, Torres-Sánchez C, Lynch CD, Gutiérrez-Pérez JL, Torres-Lagares D (2014). Outcome of single immediate implants placed in postextraction infected and non-infected sites, restored with cemented crowns: a 3-year prospective study. J Dent.

[18] Crippa R, Aiuto R, Guardincerri M, Peñarrocha Diago M, Angiero F. Effect of laser radiation on infected sites for the immediate placement of dental implants. Photobiomodul Photomed Laser Surg. 2020;38:(3)186–92. 10.1089/photob.2019.4636. 10.1089/photob.2019.4636 31429669

[19] Kakar A, Kakar K, Leventis MD, Jain G (2020). Immediate implant placement in infected sockets: a consecutive cohort study. J Lasers Med Sci.

[20] Lee J, Park D, Koo KT, Seol YJ, Lee YM (2018). Comparison of immediate implant placement in infected and non-infected extraction sockets: a systematic review and meta-analysis. Acta Odontol Scand.

[21] Renvert S, Persson GR, Pirih FQ, Camargo PM (2018). Peri-implant health, peri-implant mucositis, and peri-implantitis: case definitions and diagnostic considerations. J Periodontol.

[22] Berberi AN, Tehini GE, Noujeim ZF, Khairallah AA, Abousehlib MN, Salameh ZA (2014). Influence of surgical and prosthetic techniques on marginal bone loss around titanium implants. Part I: immediate loading in fresh extraction sockets. J Prosthodont.

[23] Kühl S, Zürcher S, Zitzmann NU, Filippi A, Payer M, Dagassan-Berndt D (2016). Detection of peri-implant bone defects with different radiographic techniques—a human cadaver study. Clin Oral Implants Res.

[24] Sirin Y, Horasan S, Yaman D, Basegmez C, Tanyel C, Aral A, Guven K (2012). Detection of crestal radiolucencies around dental implants: an in vitro experimental study. J Oral Maxillofac Surg.

[25] Verma SK, Maheshwari S, Singh RK, Chaudhari PK (2012). Laser in dentistry: an innovative tool in modern dental practice. Natl J Maxillofac Surg.

[26] Matulić N, Bago I, Sušić M, Gjorgievska E, Kotarac Knežević A, Gabrić D (2019). Comparison of Er:YAG and Er, Cr:YSGG laser in the treatment of oral leukoplakia lesions refractory to the local retinoid therapy. Photobiomodul Photomed Laser Surg.

[27] Dereci Ö, Hatipoğlu M, Sindel A, Tozoğlu S, Üstün K. The efficacy of Er,Cr:YSGG laser supported periodontal therapy on the reduction of peridodontal disease related oral malodor: a randomized clinical study. Head Face Med. 2016;12(1):20. 10.1186/s13005-016-0116-y. 10.1186/s13005-016-0116-y PMC4855779 27145828

[28] Hakki SS, Korkusuz P, Berk G, Dundar N, Saglam M, Bozkurt B (2010). Comparison of Er, Cr:YSGG laser and hand instrumentation on the attachment of periodontal ligament fibroblasts to periodontally diseased root surfaces: an in vitro study. J Periodontol.

[29] Martins M, Carvalho M, Pina-Vaz I, Capelas J, Martins M, Gutknecht N (2014). Outcome of Er, Cr:YSGG laser-assisted treatment of teeth with apical periodontitis: a blind randomized clinical trial. Photomed Laser Surg.

[30] Waasdorp JA, Evian CI, Mandracchia M (2010). Immediate placement of implants into infected sites: a systematic review of the literature. J Periodontol.

[31] Crippa R, Aiuto R, Dioguardi M, Peñarrocha-Diago M, Peñarrocha-Diago M, Angiero F. Laser therapy for infected sites and immediate dental implants in the esthetic zone: a case report and review of literature. Case Rep Dent. 2020. 10.1155/2020/2328398. 10.1155/2020/2328398 PMC6973183 31970001

[32] Romeo E, Ghisolfi M, Carmagnola D (2004). Peri-implant diseases. A systematic review of the literature. Minerva Stomatol.

[33] Dent CD, Olson JW, Farish SE, Bellome J, Casino AJ, Morris HF (1997). The influence of preoperative antibiotics on success of endosseous implants up to and including stage II surgery: a study of 2,641 implants. J Oral Maxillofac Surg.

[34] Esposito M, Grusovin MG, Talati M, Coulthard P, Oliver R, Worthington HV (2008). Interventions for replacing missing teeth: antibiotics at dental implant placement to prevent complications. Cochrane Database Syst Rev.

[35] Barone A, Marchionni FS, Cinquini C, Panattoni AC, Toti P, Marconcini S (2017). Antibiotic treatment to prevent postextraction complications: a monocentric, randomized clinical trial. Preliminary outcomes. Minerva Stomatol.

[36] Rabel A, Kohler SG (2006). Microbiological study on the prognosis of immediate implant and periodontal disease. Mund Kiefer Gesichtschir.

